# Automated Online Direct mRNA Sequence Mapping Using Partial RNase T1 Digests

**DOI:** 10.1021/acs.analchem.5c08110

**Published:** 2026-03-23

**Authors:** Jessica S. Dale, Emma N. Welbourne, Caroline A. Evans, Thomas C. Minshull, Alexander B. Schwahn, Fiona Rupprecht, Ken Cook, Kate A. Loveday, Zoltan Kis, Mark J. Dickman

**Affiliations:** † School of Chemical, Materials and Biological Engineering, 7315 University of Sheffield, Sheffield S1 3JD, U.K.; ‡ 10289 Thermo Fisher Scientific (Schweiz) AG, Neuhofstrasse 11, Reinach 4153, Switzerland; § Thermo Fisher Scientific, Stafford House, 1 Boundary Park, Hemel Hempstead HP2 7GE, U.K.; ∥ Department of Chemical Engineering, Imperial College London, South Kensington Campus, London SW7 2AZ, U.K.

## Abstract

Mass spectrometry-based approaches have emerged as powerful tools for the analysis of a wide range of critical quality attributes of mRNA medicines, including sequence identity, 5′ capping efficiency, and 3′ poly­(A) tail length and heterogeneity. These critical quality attributes can impact the quality, safety, and efficacy of mRNA medicines. In this study, we have utilized online partial RNase T1 digests in conjunction with two-dimensional liquid chromatography mass spectrometry (2D LC–MS) for the direct sequence mapping of mRNA. Automated online partial RNase T1 digests are performed in conjunction with ion-pair reversed-phase liquid chromatography. No sample or solvent manipulation is required following online RNase digestions, demonstrating the simplicity of the method. High-resolution tandem mass spectrometry was used to identify the corresponding oligoribonucleotides and generate mRNA sequence maps. High sequence coverage (93–99%) for eGFP mRNA was obtained in <60 min based only on unique oligoribonucleotide identifications. Moreover, the online partial RNase T1 digests result in controlled, fully automated and reproducible mRNA digests, enabling high-throughput, direct mRNA sequence mapping studies. The online partial RNase digests offer significant advantages over existing methods for rapid, automated mRNA identity testing. Furthermore, precise control of the digest conditions via flow rate and temperature of the online RNase T1 digest, enables multiattribute monitoring of 5′ capping efficiency, mRNA sequence mapping, and 3′ poly­(A) tail length and heterogeneity in a fully automated 2D LC–MS workflow.

## Graphical abstract

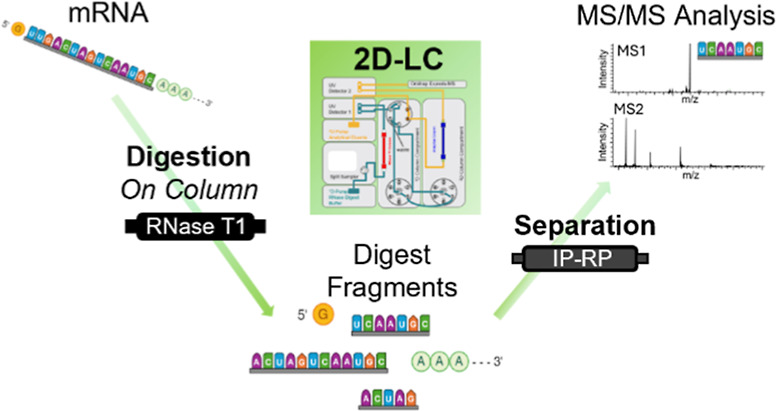

## Introduction

mRNA technology has emerged as a powerful new platform for developing mRNA medicines, exemplified by the development and approval of two highly efficacious vaccines based on mRNA sequences encoding for a modified version of the SARS-CoV-2 spike protein.^1^ ,^2^ Furthermore, RNA-based approaches have potential for treatments beyond vaccines and infectious diseases as therapeutics for cancer, metabolic disorders, cardiovascular conditions, and autoimmune diseases.^3–5^ mRNA medicines work by translating exogenous mRNA into the target protein.^6^ During the enzymatic manufacturing process of mRNA medicines, incomplete mRNA products can be generated in conjunction with other potential impurities such as double-stranded RNA.^7,8^

Comprehensive analytical methods are crucial for the characterization of mRNA medicines and supporting manufacturing process development. The implementation of validated analytical methods is required to address the specific needs of each stage of clinical development, to fulfill regulatory submission obligations, and to ensure rigorous quality control of licensed products. At present, there remains a substantial demand for the advancement of analytical technologies capable of providing more detailed and reliable characterization of RNA-based therapeutics.

Liquid chromatography interfaced with tandem mass spectrometry (LC–MS/MS) has emerged as a powerful tool for the analysis and characterization of mRNA medicines.^9,10^ mRNA identity is a critical quality attribute (CQA) inherent to drug efficacy.^11–13^ Direct mRNA sequence mapping by LC–MS/MS has emerged as a powerful, orthogonal approach to the more conventional Sanger or next-generation sequencing methods. This sequencing method is a direct approach, providing an unbiased and accurate evaluation of the mRNA primary sequence and its modifications without the need for conversion to cDNA or amplification.^9,10,14^

Several alternative workflows have been established for mRNA characterization using direct mRNA sequence mapping in conjunction with mass spectrometry. Sequence mapping approaches based on site-specific ribonucleases (RNases) have been developed to confirm the identity, primary sequence, and chemical modifications of in vitro transcribed (IVT) mRNA.^15–20^ However, the characterization of large mRNA by LC–MS/MS remains technically demanding, largely due to the limited availability of robust analytical and computational tools. Commonly used high-frequency RNases, such as RNase T1 and RNase A, typically produce short oligoribonucleotide fragments that cannot be uniquely assigned to the mRNA sequence. In contrast, enzymes such as the *E. coli* interferase MazF generate large unique fragments that are often difficult to confidently identify based on MS/MS spectra.^17,21–23^

To address these challenges, novel sequence mapping strategies have been explored, including partial T1 digestions,^16^ parallel digestions using multiple RNases,^17,25^ and the use of alternative nucleases such as human RNase 4,^18^ thereby improving sequence coverage. Goyon et al. recently introduced an online RNase digestion platform designed to streamline and automate mRNA analysis.^24^ Parallel RNase digestions in the first dimension were interfaced with hydrophilic interaction liquid chromatography (HILIC). Using this method, sequence coverages ranging from 5.8 to 51.5% with RNase T1 and 3.5 to 19.3% with RNase A were obtained from unique digestion products of five model mRNAs. We have previously established and applied a direct mRNA sequence mapping strategy employing partial RNase T1 digestion combined with ion-pair reversed-phase LC–MS (IP-RP LC–MS).^16^

Furthermore, we have developed novel visualization tools that integrate oligoribonucleotide identifications from multiple LC–MS/MS data sets, enabling the use of extensive overlapping fragments and complementary partial RNase digests to enhance, streamline, and optimize mRNA sequence mapping.^25^

In this study, we utilized online partial RNase T1 digests in conjunction with IP-RP LC-MS for the direct sequence mapping of mRNA. Automated online partial digests were performed prior to IP-RP LC-MS/MS analysis. A fully automated 2D LC–MS workflow enabled high sequence coverage of unmodified and N1-methylpseudouridine (m1Ψ)-modified mRNAs using only unique oligoribonucleotide fragments. Furthermore, this method enables multiattribute monitoring of the 5′ capping efficiency, mRNA identity, and characterization of the 3′ poly­(A) tail length and heterogeneity.

## Experimental Section

### Chemicals

Water (UHPLC MS grade, Thermo Scientific), acetonitrile (UHPLC MS grade, Thermo Scientific), 1,1,1,3,3,3-hexafluoro-2-propanol (HFIP, >99.8% Fluka LC-MS grade), triethylamine (TEA, 99.7% extrapure Fisher Scientific), and triethylammonium acetate (TEAA, pH 7.4, HPLC grade, Glen Research).

### IVT and Purification of mRNA

mRNA synthesis via IVT was performed using linearized plasmid DNA (GenScript), DNA-dependent RNA polymerase of T7 bacteriophage (Roche), and ATP, CTP, GTP, and UTP/(m1Ψ UTP) (Roche) in an equimolar ratio at 10 mM concentration. The reaction mixture was further supplemented with the standard reaction buffer recommended by the enzyme manufacturer. Inorganic pyrophosphatase (Roche) at 2.9 × 10^–3^ mM was added to the reaction mixture to prevent magnesium pyrophosphate precipitation.

RNase inhibitor (Roche) was added at 2.1 × 10^–4^ mM to maintain an RNase-free environment in the reaction mixture. The reaction was incubated at 37 °C for 2 h. Following IVT, template DNA was removed by the addition of DNase I, and RNA was purified using silica columns, as previously described. RNA concentrations were determined using a NanoDrop 2000c spectrophotometer (Thermo Fisher Scientific) by absorbance at 260 nm normalized to a 1.0 cm (10.0 mm) path. mRNA encoding SARS-CoV-2 Spike protein (CSP) (4286 nts) and eGFP (930 nts) was prepared using a DNA template containing the open reading frame flanked by the 5′ and 3′ untranslated regions (UTR) and a poly­(A) tail. The size and integrity of the mRNA were assessed using capillary electrophoresis (see Figure S1).

### 2D HPLC

A Vanquish Loop Heart-Cut 2D-LC System (Thermo Fisher Scientific) was used with the following modules: Split Sampler FT, Vanquish Dual Pump F, Vanquish Binary Pump F, two Vanquish Column Compartments H (6-position 7-port valves are used and depicted in Figure 1), and two Vanquish VWD-F UV detectors set to acquire at 260 nm. The 2D-LC system was coupled with either an Orbitrap Exploris 240 Mass Spectrometer or an Orbitrap Exploris 480 Mass Spectrometer (Thermo Fisher Scientific). 2D LC was performed using a SMART Digest RNase T1 Column of 2.1 mm × 30 mm (Thermo Fisher Scientific) in conjunction with either a DNAPac RP 4 μm 2.1 mm × 100 mm (Thermo Fisher Scientific) or an XBridge Premier Oligo BEH C18 130 Å 2.5 μm 2.1 mm × 100 mm column (Waters).

**Figure 1:**
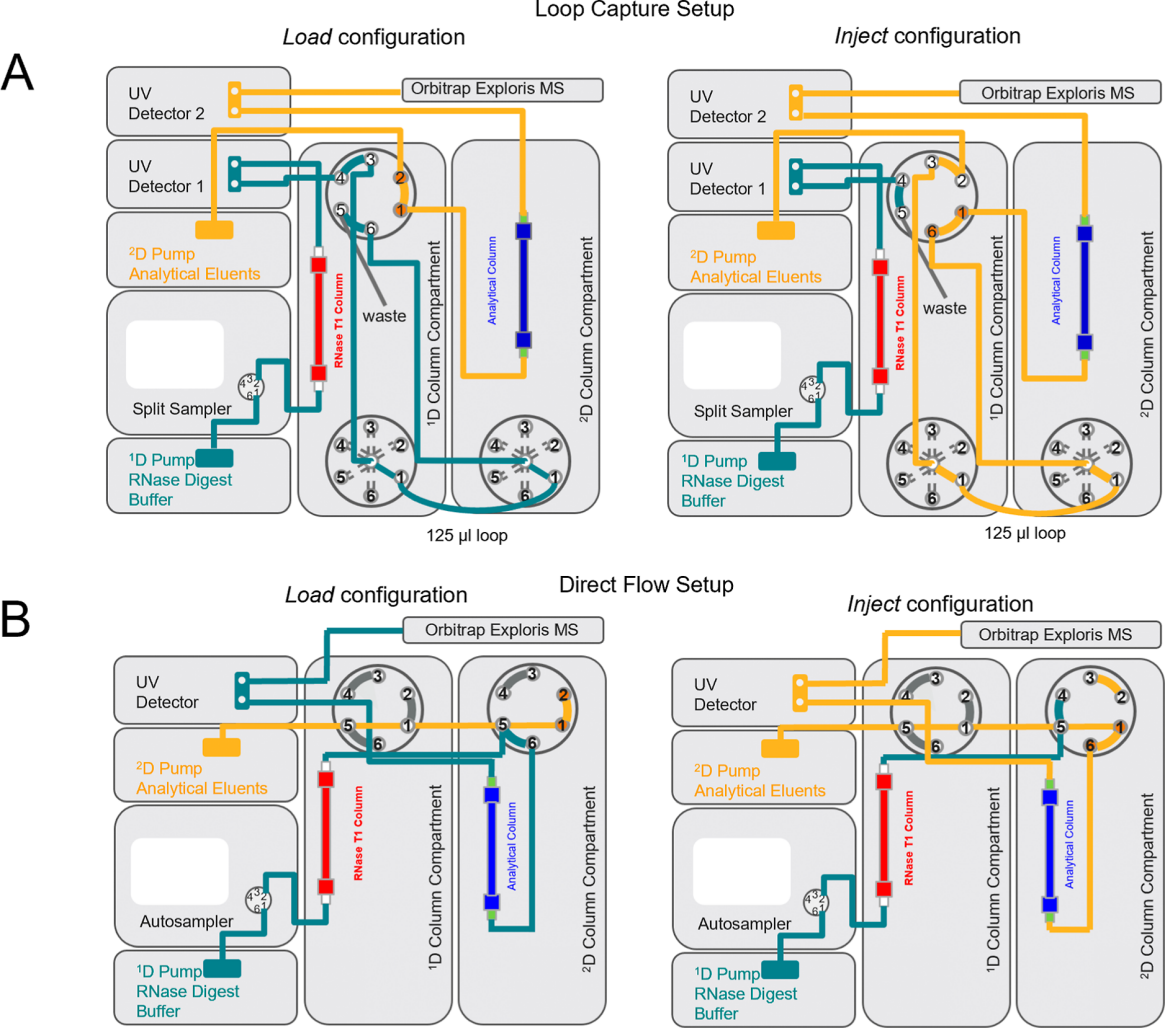
Schematic illustration of the 2D LC configuration. (A) 2D LC configuration for loop capture of the mRNA digest. (B) 2D LC configuration for direct flow of the mRNA digest. The flow paths for the “load” and “inject” configurations are highlighted.

RNase T1 digest buffer was 100 mM triethylammonium acetate (TEAA, pH 7.4). For the analytical second dimension (2D) methods, mobile phase A was 0.2% TEA, 50 mM HFIP in water, and mobile phase B was 0.2% TEA, 50 mM HFIP with 20% ACN. mRNA samples were prepared in 100 mM TEAA. The analytical 2D column compartment and preheater were set to 60 °C for all methods.

### Loop Capture Methods (Partial Digest)

Partial RNase T1 digests were performed across a range of different flow rates, 10–70 μL/min, across varying temperatures, 5–45 °C. The Loop Capture instrument setup is depicted in Figure 1. The upper six-port cut valve on the first dimension (1D) column compartment was used to collect digest fragments in a 125 μL loop (position 1_2) and inject fragments onto the analytical column (position 6_1). The 125 μL loop was configured in the flow path between the two lower array valves, at position 1, on the 1D column compartment, and the 2D column compartment.

In order to derive the valve switch times, a mixture of 9 DNA oligonucleotides varying from 8 to 40 nucleotides in length was injected at flow rates of 10, 20, 30, and 50 μL/min. The retention time of the absorbance peak at 260 nm for the oligonucleotide mixture on the 1D Variable Wavelength Detector (VWD) was recorded for each flow rate. This retention time was added to the time taken for the peak of digest fragments to reach halfway across the 125 μL loop at the given flow rate using the known volume of tubing on the HPLC instrument. This was then set as the valve switching time. For experiments where the flow rate was 40, 60, and 70 μL/min, the valve switching time was derived from extrapolation of the existing retention time data.

Analytical separations were performed by using the following gradients. Gradient 1: 0–18% MPB using a nonlinear gradient (curve 4) over 40 min. Gradient 2: 0–20% MPB using a linear gradient (curve 5) of 40 min. Gradient 3: 0–20% MPB using a linear gradient (curve 5) over 50 min using a flow rate of 250 μL/min and UV detection at 260 nm. A DNAPac RP 4 μm 2.1 × 100 mm column was used as the analytical column.

### Direct Flow 2D LC (Partial Digest)

Flow was delivered directly from the RNase T1 column to the analytical column via a 6 port cut valve for the first 10 min. At 10 min, the 6 port cut valve was switched to deliver the gradient across the analytical column. The flow rate through the digest column was 50 μL/min for unmodified eGFP mRNA, 30 μL/min for m1Ψ eGFP mRNA, 20 μL/min for unmodified CSP mRNA, and 10 μL/min for m1Ψ CSP mRNA. The digest column temperature was 25 °C for unmodified and m1Ψ eGFP mRNA and m1Ψ CSP mRNA and 20 °C for unmodified CSP mRNA. The analytical column flow rate was raised from 50 to 250 μL/min over a period of 2 min. Analytical separations were performed using the following gradients. Gradient 1: 0–18% MPB using a nonlinear gradient (curve 4) over 48 min. Gradient 2: 0–20% MPB using a nonlinear gradient (curve 5) over 51 min using a flow rate of 250 μL/min and UV detection 260 nm. The flow rate of analytical eluents was held at 50 μL/min during digestion before being raised to 250 μL/min at the start of the gradient. A DNAPac RP 4 μm 2.1 × 100 mm column was used as the analytical column.

### Mass Spectrometry

Data-dependent acquisition (DDA) was applied in full-scan negative mode, scanning from 450 to 3000 *m*/*z*. The MS1 resolution was set to 120,000, and the normalized automatic gain control (AGC) target was 200%. MS1 ions were selected for higher-energy collisional dissociation (HCD). The RF lens was set to 70%. The MS2 resolution was set at 30,000 with the AGC target of 100%, an isolation window of 3 *m*/*z*, a scan range of 150–2000 *m*/*z*, and HCD Collision Energies set to step at 17%, 20%, and 23%.

### LC–MS/MS Data Analysis

Data analysis was performed in BioPharma Finder v5.2 (BPF, Thermo Fisher Scientific), using the Oligonucleotide Analysis module with “Enable Automatic Parameter Values” selected for component detection. To identify large fragment ions, the maximum oligonucleotide mass was set to 30,000 Da, minimum confidence at 0.90, and mass accuracy at 5 ppm. The ribonuclease selection was set to RNase T1, with default specificity (G-). The specificity level was set at “high”. The phosphate location was set at “3′-cyclic”. Phosphorylation was set as a variable modification of the 3′ terminal in the sequence manager containing the RNA sequence. For data processing and review additional filters were included: “Identification” = “does not contain nonspecific”, “does not contain nonunique”; “Mod” = “does not contain None”; “Nonunique Seq” = “≤ 1”; “Δppm” = “≤ 20”, “≥ −20”; “Conf. Score” = “≥90”; “Best ASR” = “≤ 2.0”; “ID Type” = “contains MS2”; “Mono Mass Exp.” = “>0”. Data visualization and generation of linear and spiral sequence maps were performed using in-house software, which is freely available online (13). All oligonucleotide identifications from BPF are shown in the Supporting Information (Table S1). Random RNA sequences of the same length and GC content were included in the sequence manager in addition to the correct RNA sequence. % Sequence coverage of random RNA sequences is provided in the Supporting Information (Tables S2–S6).

## Results and Discussion

Sequence mapping of mRNA using online partial RNase T1 digests in conjunction with 2D LC–MS.

### Loop Capture

Initial work focused on the optimization of online partial RNase T1 digests in conjunction with IP-RP LC-MS/MS. The 2D HPLC was set up without the requirement for trap columns or Active Solvent Modulation (see Figure 1A). Using this 2D LC set up, the mRNA is directly injected onto the RNase T1 column in the first dimension, prior to collection in a 125 μL internal loop positioned in the flow path between the RNase T1 column and the analytical column (see Figure 1A). Following collection of the mRNA digest into the middle of the loop, the digested mRNA was injected onto the analytical column prior to gradient elution using IP-RP HPLC interfaced with mass spectrometry. The use of triethylammonium acetate as the RNase digest buffer in the first dimension ensured the retention of the mRNA digest fragments on the analytical reversed phase column. Second dimension separations were subsequently performed using TEA/HFIP mobile phases. Oligoribonucleotide fragments were identified using high-resolution accurate mass and tandem MS. mRNA sequence coverage was determined using only unique oligoribonucleotide fragment identifications, and the percentage coverage was determined from the mRNA sequence (not including the poly­(A) tail).

Optimization of the online partial RNase T1 digestions was simply performed by varying the temperature and flow rate (residence time) in the RNase T1 column. The corresponding UV chromatograms, linear mRNA sequence maps, and overall sequence coverages for the analysis of eGFP mRNA and an mRNA encoding the SARS-CoV-2 spike protein (CSP mRNA) across varying temperatures (5–45 °C) are shown in Figure 2. Varying the temperature of the RNase T1 column while keeping the flow rate constant (50 μL/min) in the first dimension shows that as the temperature is increased, there is a decrease in the number and abundance of larger oligoribonucleotide fragments. This is evident by the elution peaks in the chromatograms and the identified fragments in the sequence maps (see Figure 2). As the temperature is increased to 35/45 °C, many of the larger oligonucleotide fragments are further digested to shorter fragments (less missed cleavages), resulting in a reduction in the number of overlapping oligoribonucleotide fragments identified. For eGFP mRNA, the highest sequence coverage (>94%) was obtained at 5–25 °C. For CSP mRNA, the highest sequence coverage (>60%) was obtained at 15/25 °C. Furthermore, high sequence coverages with no or low sequence matches against random control RNA sequences were obtained, demonstrating the specificity of the analytical workflow in conjunction with the parameters used for RNA sequence mapping (see Tables S2 and S3).

**Figure 2:**
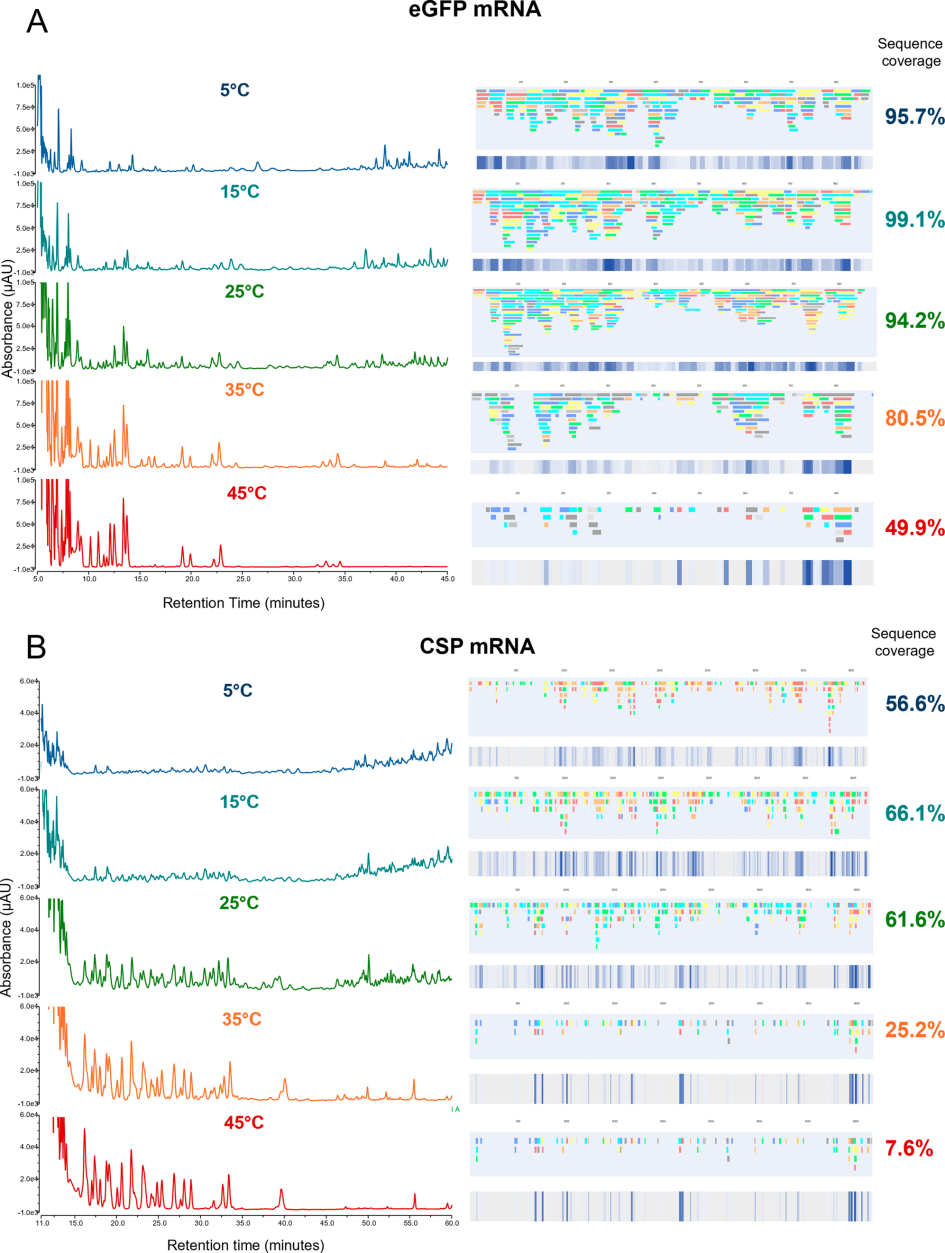
mRNA sequence mapping using online partial RNase T1 digests at different temperatures. IP-RP UV chromatograms of the online digests at varying column temperatures and linear sequence maps generated from the oligoribonucleotide fragments identified via LC–MS/MS are shown for (A) eGFP mRNA (10 μg) and (B) CSP mRNA (18 μg). Overall, mRNA sequence coverage based on unique oligoribonucleotides is highlighted.

Further optimization was performed by varying the flow rate of the RNase T1 column while keeping temperature constant (25 °C for eGFP mRNA and 20 °C for CSP mRNA) (see Figure 3). As expected, as the flow rate is increased (residence time decreased), there is an increase in the relative abundance and number of larger oligoribonucleotide fragments, which elute later in the chromatography (see Figure 3). As flow rate is decreased, many of the larger oligonucleotide fragments are further digested to shorter fragments (less missed cleavages), resulting in a reduction in the number of overlapping oligoribonucleotide fragments identified. For eGFP mRNA, the highest sequence coverage (>90%) was obtained between 40 and 70 μL/min. For CSP mRNA, the highest sequence coverage (>80%) was obtained between 30 and 50 μL/min (see Figure 3 and Tables S4–S5).

**Figure 3:**
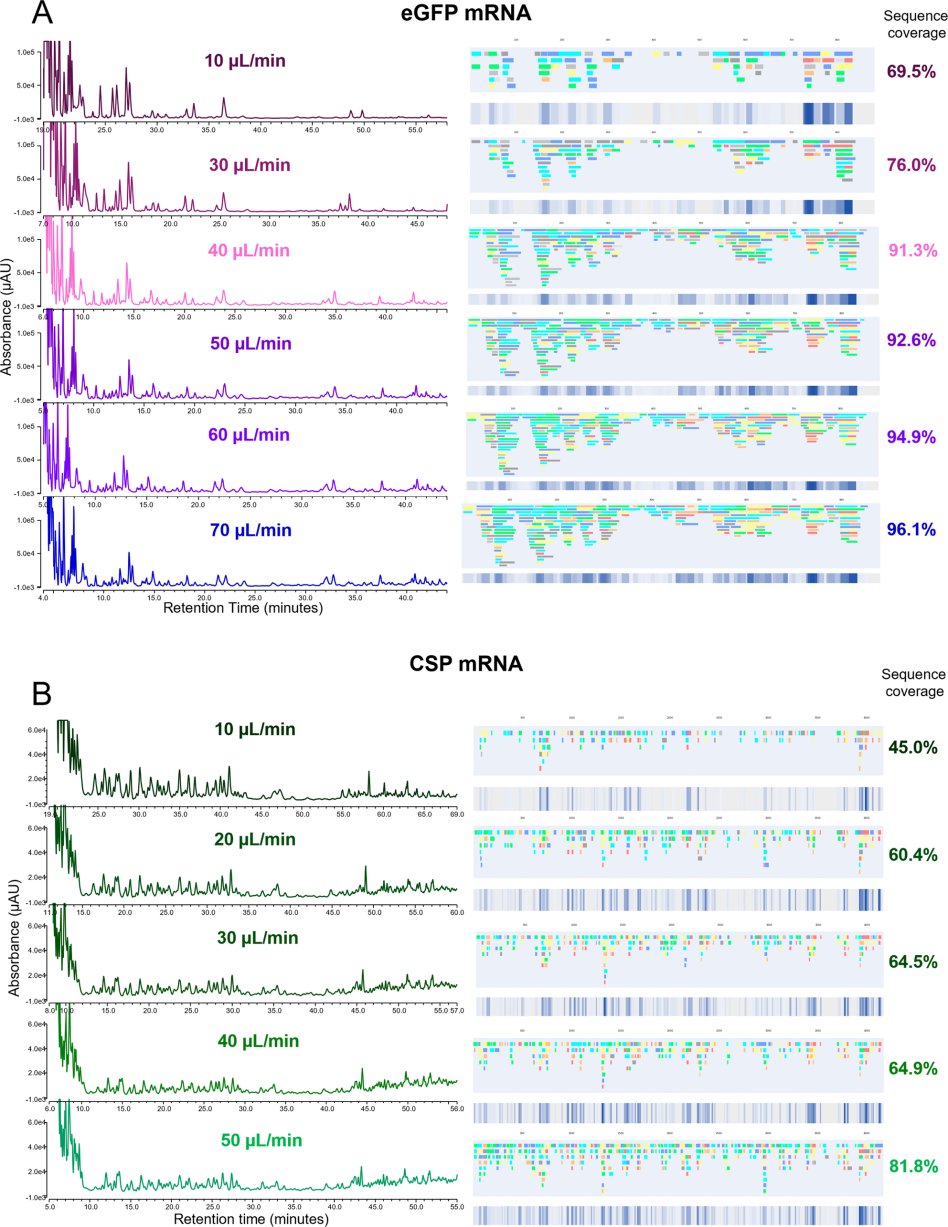
mRNA sequence mapping using online partial RNase T1 digests at different flow rates. IP-RP UV chromatograms of the online digests at varying flow rates and linear sequence maps generated from the oligoribonucleotide fragments identified via LC–MS/MS are shown for (A) eGFP mRNA (10 μg) and (B) CSP mRNA (18 μg). Overall, mRNA sequence coverage based on unique oligoribonucleotides is highlighted.

The ability to combine multiple mRNA digestions across varying conditions, e.g., temperature and flow rates, can be used to further increase mRNA sequence coverage for challenging long mRNAs or RNAs with a high degree of secondary structure. The sequence mapping analysis of CSP mRNA at 5 °C, 25 °C, and 45 °C is shown in Figure 4. By combining the oligoribonucleotide fragments identified in each of the different temperatures, the overall sequence coverage increases, and regions of the mRNA where no oligoribonucleotides are identified are reduced (see Figure 4). Individually, the digests at 5 °C, 25 °C, and 45 °C produce 7.6%, 56.6%, and 61.6% sequence coverage, respectively, whereas in combination this is increased to 78%.

**Figure 4:**
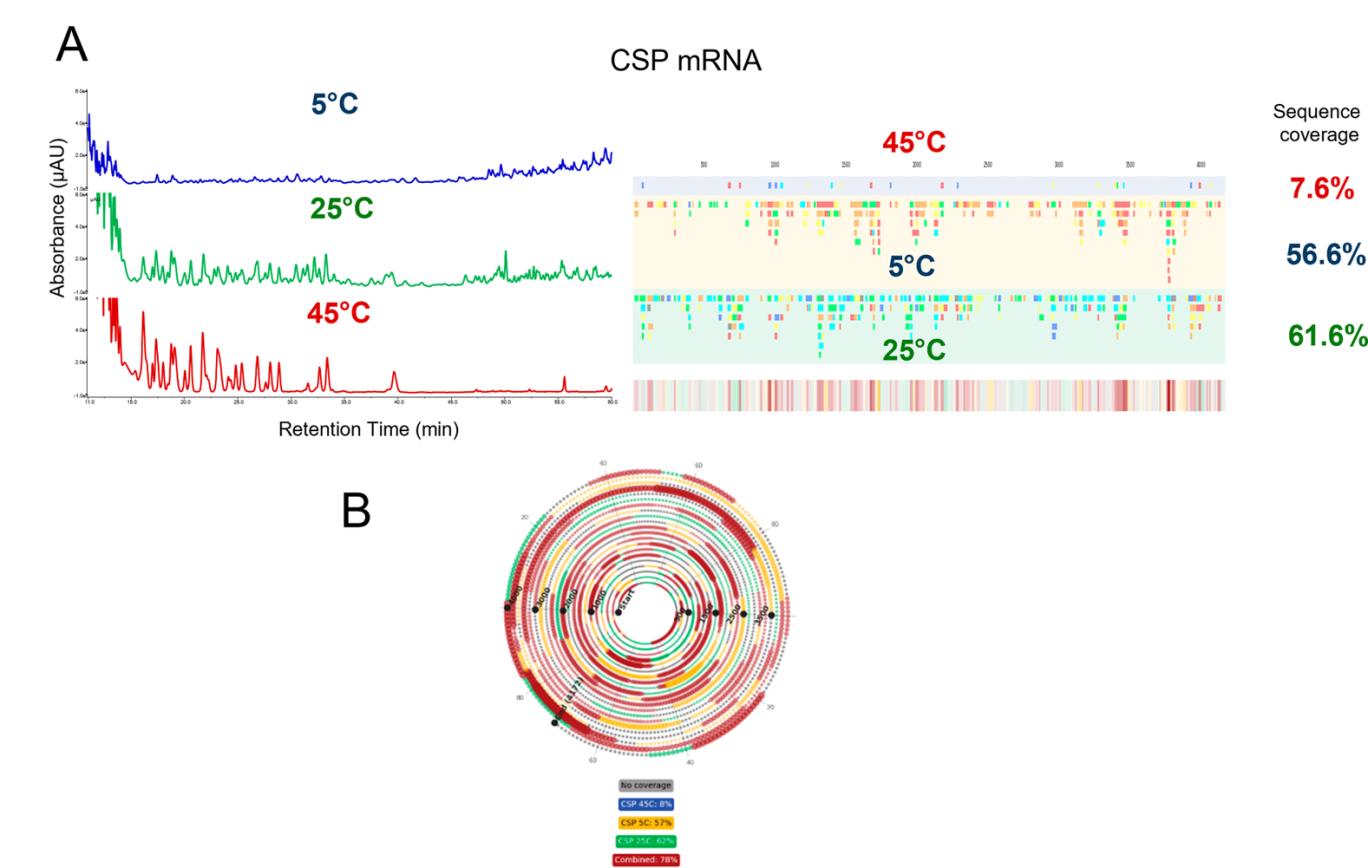
LC–MS/MS analysis of combined online RNase T1 partial digests of mRNA. (A) IP-RP UV chromatograms of the online partial RNase T1 digests of CSP mRNA at varying temperatures and linear sequence maps generated from the oligoribonucleotide fragments identified via LC–MS/MS. (B) Spiral mRNA sequence map generated from the combined mRNA digests. Fragments generated at 5 °C are mapped in yellow, fragments generated at 25 °C are mapped in green, fragments generated at 45 °C are mapped in blue, and overlapping fragments are mapped in red. Areas with no coverage are marked in gray. Overall, % mRNA sequence coverages based on unique oligoribonucleotides are highlighted.

These results demonstrate the ability to rapidly optimize partial RNase T1 digests to maximize mRNA sequence coverage by simply varying the temperature and flow rate across the online RNase T1 column. This generates the optimum length and number of overlapping unique oligonucleotide fragments for identification using LC–MS/MS in conjunction with HCD fragmentation. The ability to rapidly alter the mRNA digest conditions in an automated, high-throughput manner enables further insight into the potential higher order structure of the mRNA. Furthermore, the ability to combine multiple digestions across varying temperature and flow rates can be used to further increase mRNA sequence coverage for challenging long mRNAs or those with potentially high degrees of secondary structure. It is interesting to note that eGFP mRNA sequence coverage decreases under conditions using lower flow rates or higher temperatures (increased digestion) with a corresponding loss of unique oligoribonucleotide fragments, predominantly in specific regions of the mRNA sequence. The results show that for eGFP mRNA, there are regions including the 5′ end, 96–137 nts, and the 3′ end prior to the poly­(A) tail with no sequence coverage (no identified unique oligoribonucleotides) as digestion of the mRNA is increased (see Figure 2A). This suggests that the initial unique, longer length oligoribonucleotides are digested quickly to shorter, nonunique fragments, which are not shown on the mRNA sequence maps. In addition, the central region of the eGFP mRNA also shows a significant reduction in unique oligoribonucleotides as the level of digestion of the mRNA is increased. Therefore, these regions of the mRNA may have more accessible (unpaired) guanosine residues, potentially in single stranded loop structures, leading to relatively higher RNase T1 activity in these regions.

### Direct Flow 2D LC

In addition to the above loop capture 2D LC configuration, an alternative 2D LC setup was investigated using a simpler direct flow configuration (Figure 1B). The mRNA was injected onto the RNase T1 column and the resulting oligoribonucleotides flowed directly onto the analytical column. After 10 min, the oligoribonucleotide fragments were eluted from the analytical column and analyzed using MS/MS as previously described. A comparison of the LC–MS/MS mRNA sequence mapping of eGFP and CSP mRNA under the same digest conditions (flow rate and temperature) using both direct flow and loop capture is shown in Figure 5 and Table S6. No significant difference in mRNA sequence coverage for eGFP mRNA or CSP mRNA using the two different methods was observed. However, an increase in mRNA sequence coverage of eGFP m1Ψ mRNA from 78.3% to 90.7% was observed when using the loop capture approach. This was attributed to the increased signal intensity of oligoribonucleotides in this particular example using loop capture. As the mRNA digest sample is captured onto the analytical column over a 10 min period, this could impact chromatographic resolution via lack of focusing or retention of oligoribonucleotide fragments on the analytical column. Band broadening and peak distortion may result, reducing resolution and chromatographic efficiency. However, no clear difference was observed in the chromatographic resolution based on peak width at half height across a number of identified oligoribonucleotides (Figure S2).

**Figure 5:**
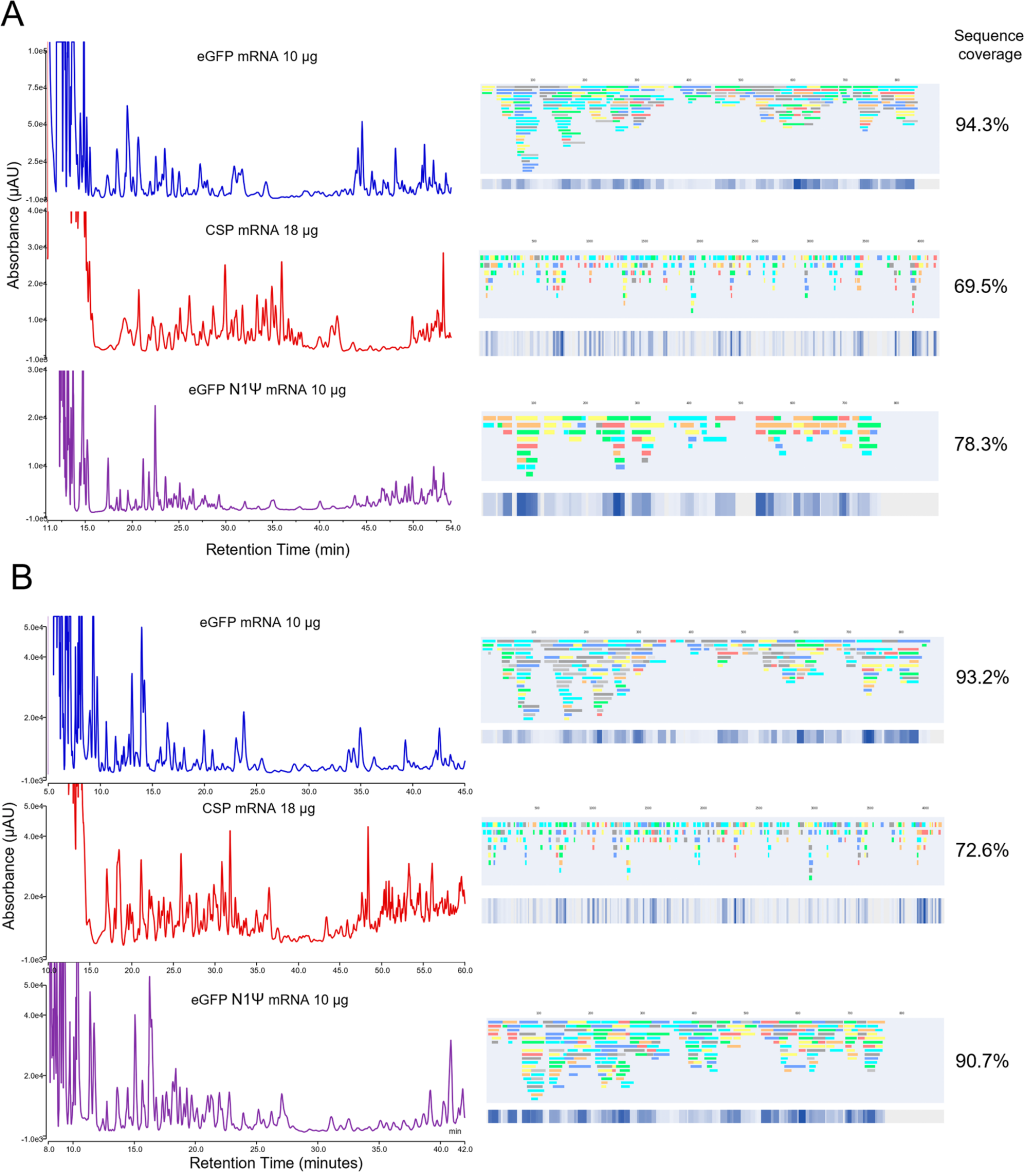
Comparative analysis of mRNA sequence mapping using loop capture and direct flow 2D LC methods. (A) mRNA sequence mapping of eGFP and CSP mRNA using direct flow 2D LC. (B) mRNA sequence mapping of eGFP and CSP mRNA using loop capture 2D LC. IP-RP UV chromatograms of the online partial RNase T1 digests and linear sequence maps generated from the identified oligoribonucleotide fragments identified via LC-MS/MS. Overall mRNA sequence coverage based on unique oligoribonucleotides are highlighted.

### Reproducibility of the Online Partial RNase T1 Digests Using 2D LC

Reproducibility of the online partial RNase T1 digests of mRNA using the online 2D LC method was demonstrated by three replicate injections of eGFP mRNA. Ten micrograms of eGFP mRNA was injected onto the RNase T1 column at 25 °C at a flow rate of 50 μL/min prior to LC–MS/MS analysis using the loop capture 2D LC configuration. The corresponding LC-UV chromatograms of the partial RNase T1 digests of eGFP mRNA are shown in Figure 6, demonstrating the reproducibility of the chromatograms obtained. To further examine the reproducibility of the online partial RNase T1 digests, further analysis of the sequence coverage and unique oligoribonucleotide identifications was performed.

**Figure 6:**
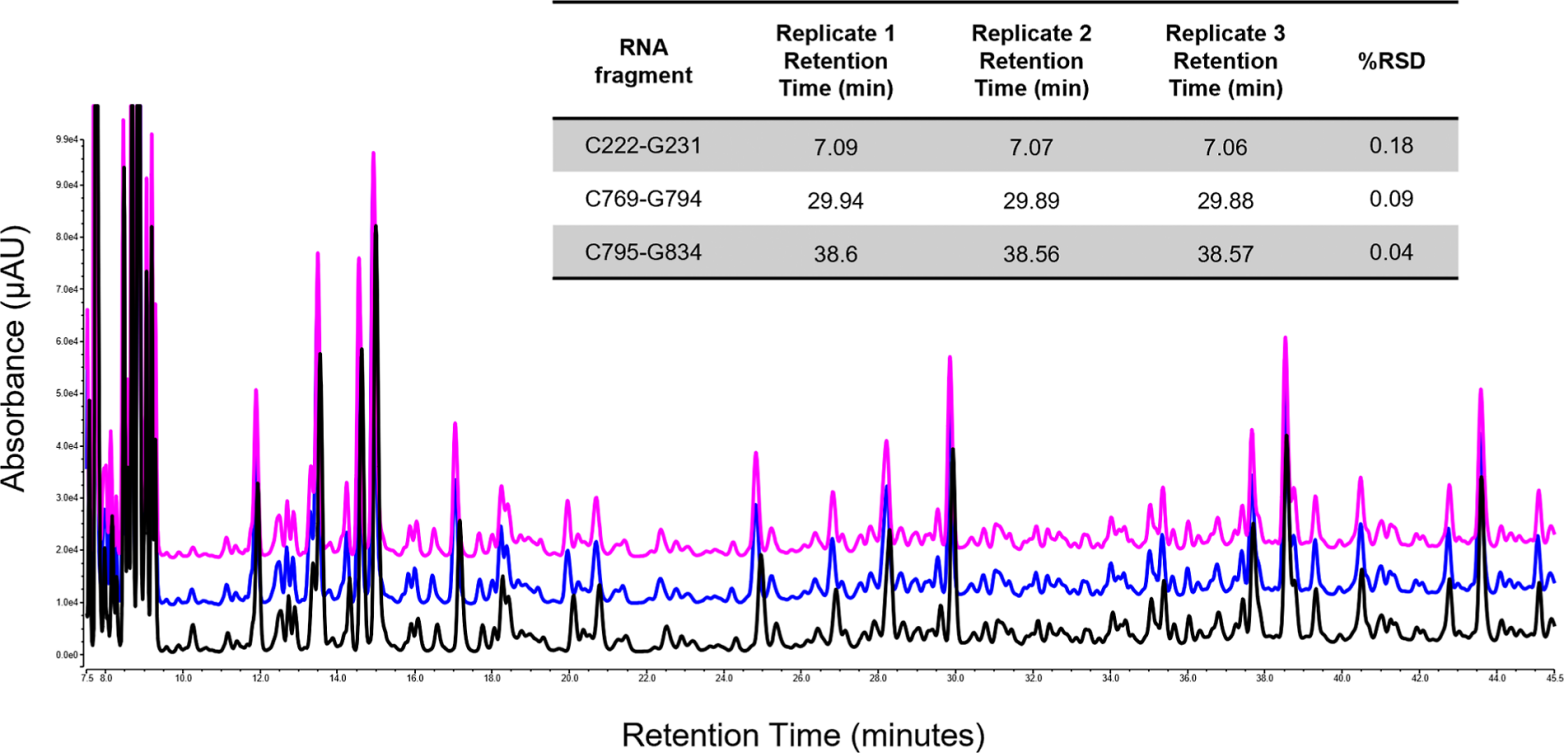
Reproducibility of partial RNase T1 digests of mRNA. IP-RP UV chromatograms of the partial RNase T1 digests of eGFP mRNA. Ten microgram of mRNA was injected onto the RNase T1 column (25 °C, flow rate 50 μL/min) prior to LC–MS/MS analysis. The number of unique oligoribonucleotides and % sequence coverage are highlighted in each replicate. The retention time and RSD of three identified unique oligoribonucleotides from the LC–MS/MS analysis across the replicates are shown for each oligoribonucleotide.

The mean sequence coverage for the eGFP replicates was 96% (relative standard deviation (RSD) 1%). These results highlight the reproducible oligoribonucleotide identifications and resulting sequence coverage across the three replicate partial RNase T1 digests. Further analysis of the retention time stability across the replicates was performed by monitoring selected identified oligoribonucleotides. The results show that the RSD of the retention time was below 0.3% for each of the oligoribonucleotides shown (see Figure 6).

### Comparison of Online vs Offline Partial RNase T1 Digests

Following optimization, we benchmarked the mRNA sequence mapping of online partial RNase T1 digests against an offline partial T1 digest using RNase T1 enzyme immobilized on magnetic beads.^16^ Varying amounts of eGFP mRNA (1 and 10 μg) were analyzed using both offline and online partial RNase T1 digests in conjunction with LC-MS/MS.

Comparative analysis of the partial RNase T1 digests performed online versus offline highlights potential differences in digest conditions. Using conditions optimized for maximum sequence coverage, the vast majority of oligoribonucleotides generated by an offline RNase T1 digest contain a 2′,3′ cyclic phosphate termini, consistent with the incomplete hydrolysis, which is expected under partial RNase T1 digest conditions^16^ (see Table S7). The results show that for the offline partial digestion of eGFP and CSP mRNA, 1% or less of identified oligoribonucleotides contain a 3′ phosphate termini. However, for the online partial digestion, a higher proportion of 3′ phosphate termini are observed: 16% for eGFP mRNA and 13% for CSP mRNA. Partial online RNase T1 digests were also performed at 5 °C: under these conditions, the mRNA was underdigested and expected only to generate 2′,3′ cyclic phosphate termini. However, 3′ phosphate termini were also identified, indicating that online partial RNase T1 digestion (under the flow conditions used in this study) results in increased hydrolysis of 2′,3′ cyclic phosphate termini to 3′ phosphate termini compared to the offline digest. The presence of both the 2′,3′ cyclic phosphate and 3′ phosphate termini could potentially increase the complexity of the mRNA digest and decrease the signal intensity of each oligoribonucleotide fragment. The average MS1 peak areas and % sequence coverage of the uniquely identified mRNA digest fragments are shown in Table S8. The results show an increase in average MS signal intensity for online digest fragments compared with offline digest fragments across the conditions used. These results are consistent with previous studies using online complete RNase digests with HILIC, where an increase in signal intensity was observed.^24^

Switching from an offline to an online digest method likely minimizes sample losses incurred during sample transfer steps prior to the LC–MS/MS analysis. Furthermore, the oligoribonucleotide fragments are immediately analyzed using LC–MS/MS following their formation during the RNase digest, minimizing potential degradation or further digestion by residual RNases prior to detection. Consequently, online partial RNase digests minimize the formation of nonspecific oligoribonucleotides that have the potential to be generated in offline digestions. Therefore, using this automated, streamlined workflow, mRNA sequence mapping can be performed using low amounts of mRNA, without compromising high sequence coverage, which is essential for researchers working on small scale mRNA production or monitoring mRNA identity during the mRNA manufacturing process.

### Online RNase T1 Digests for Multi-Attribute Monitoring

The workflow developed for direct mRNA sequence mapping (mRNA identity) can be performed under complete digest conditions for the analysis of mRNA 5′ capping efficiency and 3′ poly­(A) tail length and heterogeneity. Online RNase T1 digests using a low flow rate (5 μL/min) and a digest column temperature of 37 °C results in a complete digestion of the 5′ Capped CSP m1Ψ modified mRNA (see Figure 7A). Validation of the Cap 1 5′ cap (m7G(5′)­ppp(5′)­(2′OMeA)­pG) (Figure 7B) was based on both high-resolution accurate intact mass analysis (HRAM) and MS/MS fragmentation. 5′ Capping efficiency (86.3%) was determined based on the relative quantification of the corresponding 5′ cap and uncapped species (including triphosphate (pppGp), diphosphate (ppGp), and monophosphate (pGp)). The split 30–70 poly­(A) tail elutes at the end of the gradient, and the length and heterogeneity were determined with nucleotide resolution based on HRAM (see Figure 7C). The heterogeneity of the 70A portion of the split poly­(A) tail is shown in Figure 7D, demonstrating that the most abundant length of the poly­(A) tail was 72 nt.

**Figure 7:**
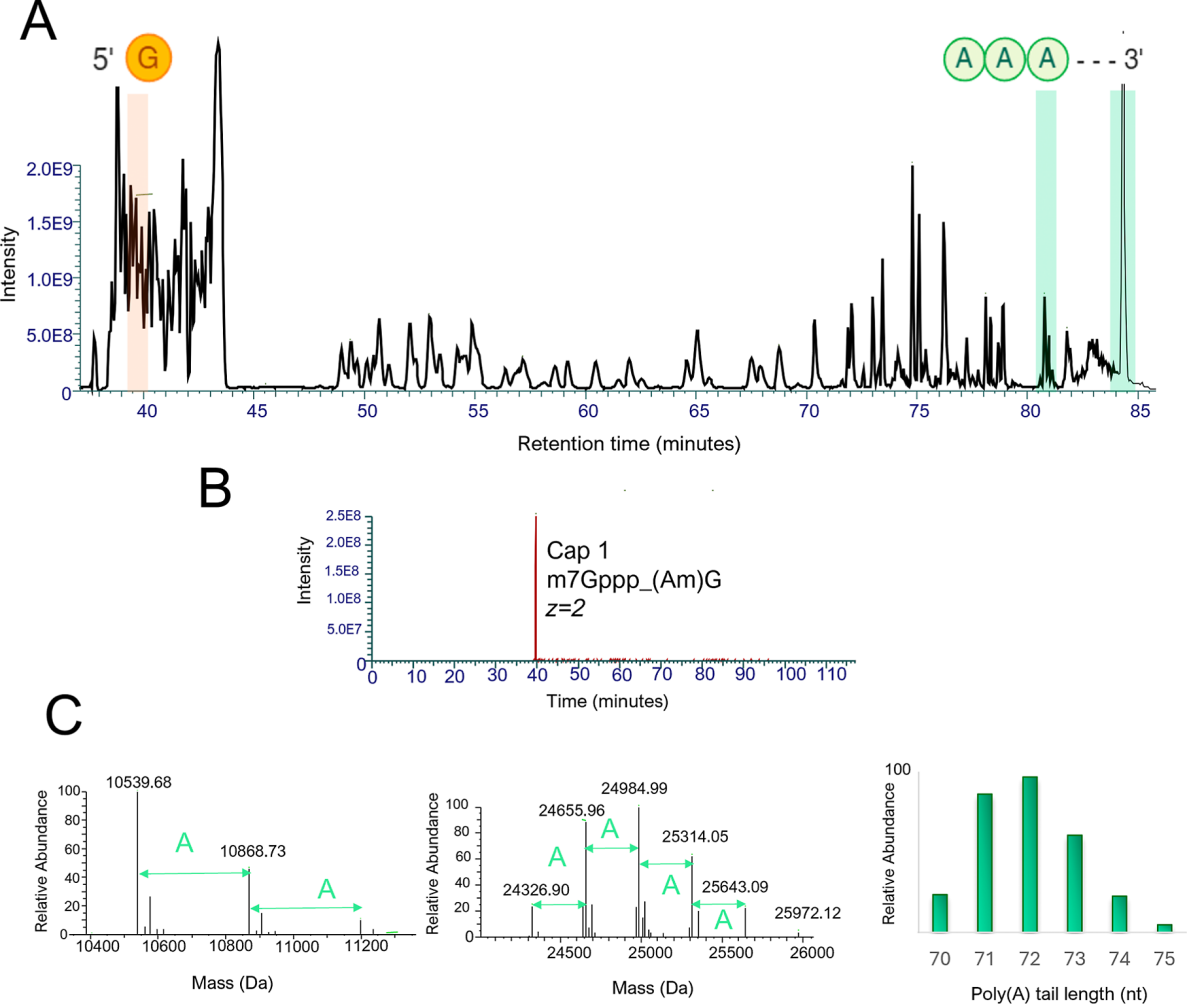
Multi-attribute monitoring of mRNA using online RNase T1 digests and LC–MS. (A) IP-RP UV chromatogram of the complete RNase T1 digest of CSP mRNA. The corresponding 5′ capped and uncapped species are highlighted in blue. The 3′ poly­(A) tail fragments are highlighted in green. (B) Extracted ion chromatograms of the 5′ Clean Cap AG fragment and 5′ uncapped triphosphate fragment. (C) Deconvoluted masses of the 3′ poly­(A) tail fragments and corresponding length and heterogeneity of the 70 nt poly­(A) fragment.

## Conclusions

Online partial RNase T1 digestion in conjunction with 2D LC MS/MS analysis was developed and utilized for direct mRNA sequence mapping. Using this 2D LC set up, the mRNA was directly injected onto the RNase T1 column in the first dimension, prior to either loop capture and injection or direct flow onto the second dimension analytical column. Optimization and precise control of the online partial RNase T1 digests were performed by varying the flow rate and temperature of the online RNase T1 digest. The online direct mRNA sequence mapping using partial RNase T1 digests developed in this study demonstrates significant advantages over current methods, including increased sequence coverage of the mRNA (based on unique oligoribonucleotide fragments), no requirement for solvent manipulation of the RNase digest fragments prior to IP-RP LC–MS analysis using fully automated 2D LC–MS equipment. High sequence coverage (>95%) of eGFP mRNA was obtained in <60 min based only on unique oligoribonucleotide identifications. High sequence coverage is more challenging for larger length mRNAs (>4000 nts) and saRNA (>10,000 nt). Strategies to achieve high sequence coverage (>80% based on unique oligoribonucleotide fragments) could include the use of complementary partial RNase digests (e.g., RNase T1/U2) and combining multiple digests over a range of different temperatures or flow rates in conjunction with an online direct mRNA sequence mapping approach. In addition, potential strategies to reduce sample complexity, such as long gradients or orthogonal 2D LC separations prior to MS analysis could also be used to further increase sequence coverage.

The online partial RNase T1 digest results in controlled, reproducible mRNA digests in a fully automated fashion, enabling high-throughput direct mRNA sequence mapping studies. In addition, the online partial RNase digests result in increased sensitivity compared to offline partial RNase digests, demonstrating the ability for fully automated mRNA sequence mapping, generating high sequence coverages from low amounts of mRNA. Furthermore, simple control of the flow rate and temperature in the online RNase T1 digest enables multi-attribute monitoring of 5′ capping efficiency, mRNA identity, and 3′ poly­(A) tail length and heterogeneity in a fully automated 2D LC workflow.

## Supplementary Material





## Notes

Capillary electrophoresis analysis of mRNA; comparison of oligoribonucleotide fragments generated in offline and online digests; oligonucleotide identifications for online mRNA digests; mRNA % sequence coverages for eGFP and CSP mRNA at different temperatures and flow rates of the RNase T1 column using Loop Capture or Direct Flow; comparison of oligoribonucleotides with 2′,3′ cyclic phosphate and 3′ phosphate termini for eGFP and CSP mRNA using online and offline partial RNase T1 digests; and comparison of average MS peak area and mRNA % sequence coverage for online and offline partial RNase T1 digests (PDF)
Identified oligoribonucleotides, from BioPharma Finder, from the partial RNase T1 digests (XLSX)
