# Supplementary material for: Automated Online Direct mRNA Sequence Mapping Using Partial RNase T1 Digests
Source: Anal Chem. 2026 Mar 23;98(13):9934–44. doi: 10.1021/acs.analchem.5c08110 (PMC13063217; doi:10.1021/acs.analchem.5c08110)
Supplement: Supplementary file 1 [file ac5c08110_si_001.pdf]

Supporting Information

Automated online direct mRNA sequence mapping using partial RNase T1 digests

[1]Jessica S. Dale, [1]Emma N. Welbourne, [1]Caroline A. Evans, [1]Thomas C. Minshull, [2]Alexander B. Schwahn, [2]Fiona A-M. Rupprecht, [3]Ken Cook, [1]Kate A. Loveday, [1,4] Zoltan Kis and [1*]Mark J. Dickman

[1]School of Chemical, Materials and Biological Engineering, University of Sheffield, Sheffield, S1 3JD, UK.

[2]Thermo Fisher Scientific (Schweiz) AG, Neuhofstrasse 11, 4153 Reinach, Switzerland.

[3]Thermo Fisher Scientific, Stafford House, 1 Boundary Park, Hemel Hempstead, HP2 7GE, UK.

[4]Department of Chemical Engineering, Imperial College London, South Kensington Campus, London SW7 2AZ, UK.

*Corresponding author m.dickman@sheffield.ac.uk

Table of Contents:

Supporting Figure S1. mRNA analysis using capillary electrophoresis

Supporting Figure S2. Comparative analysis of oligoribonucleotide fragments generated using online and offline partial RNase T1 digests.

Supporting Table S1. All oligonucleotide identifications for online mRNA sequence mapping experiments generated in BioPharma Finder.

Supporting Table S2. Comparison of mRNA % sequence coverage for eGFP mRNA analysed using online partial RNase T1 digestion across varying RNase T1 column temperatures.

Supporting Table S3. mRNA % sequence coverage for CSP mRNA analysed using online partial RNase T1 digestion across varying temperatures of the RNase T1 column.

Supporting Table S4. mRNA % sequence coverage for eGFP mRNA analysed using online partial RNase T1 digestion using varying flow rates across the RNase T1 column.

Supporting Table S5. mRNA % sequence coverage for CSP mRNA analysed using online partial RNase T1 digestion using a range of flow rates across the RNase T1 column.

Supporting Table S6. Comparison of mRNA % sequence coverage for eGFP and CSP mRNA analysed using online partial RNase T1 digest in Loop Capture and Direct Flow 2D LC.

Supporting Table S7. % abundance of oligoribonucleotide fragments identified with 2',3' cyclic phosphate and 3' phosphate termini for eGFP and CSP mRNA analysed using offline and online RNase T1 digests.

Supporting Table S8. Comparison of average MS peak area and mRNA % sequence coverage using online and offline partial RNase T1 digests

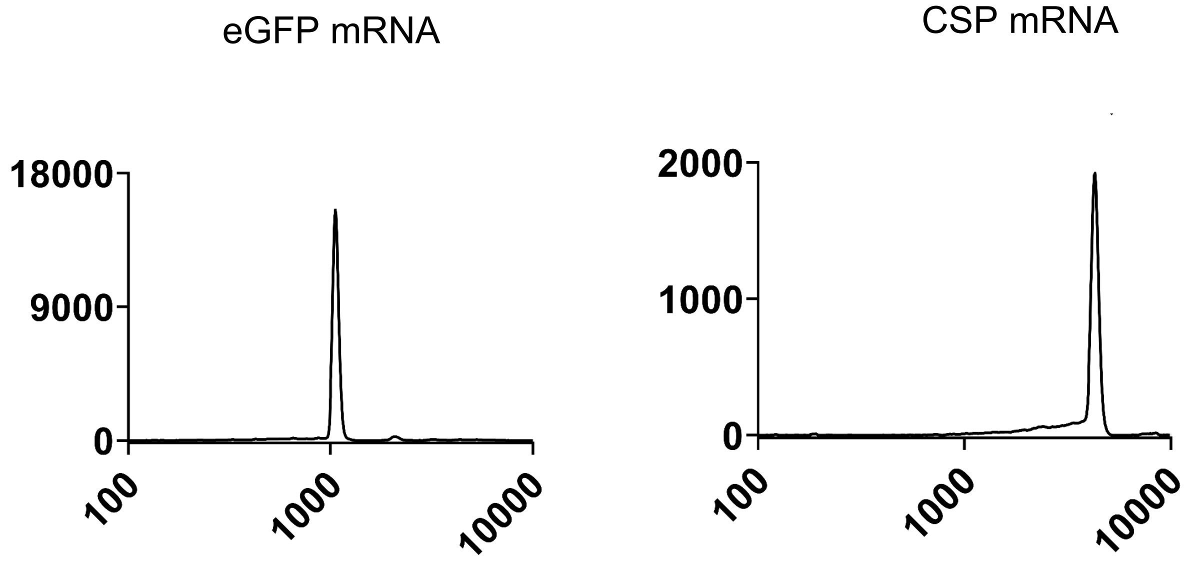

Supporting Figure S1. mRNA analysis using capillary electrophoresis. 100 ng of eGFP (930 nt) and CSP mRNA (4286 nt) was analysed on the Fragment Analyzer (Agilent). mRNA integrity was determined based on the relative peak areas of the full-length mRNA and the degraded/shorter length fragments to the left hand side of the full-length mRNA. mRNA integrity values of 95.7% and 90.8% were obtained for eGFP and CSP mRNA respectively.

A

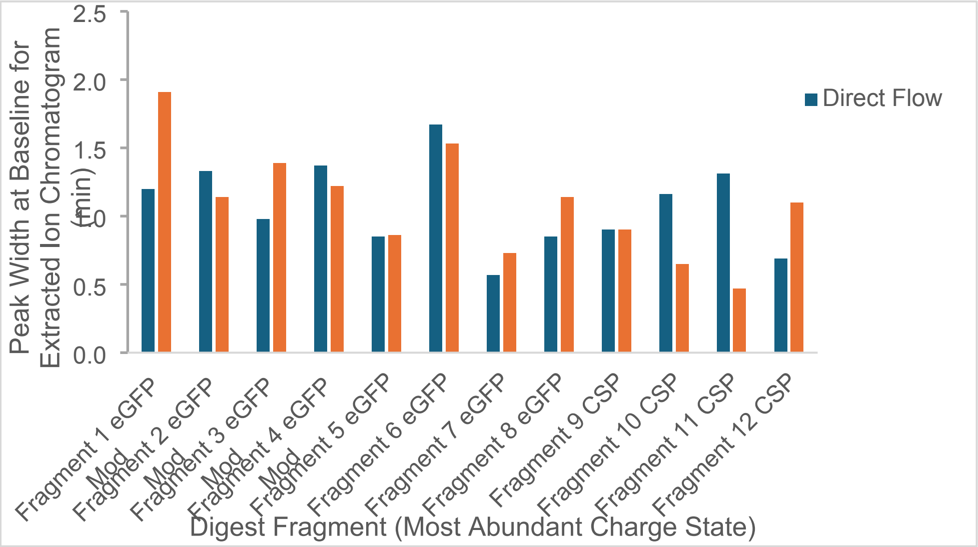

B

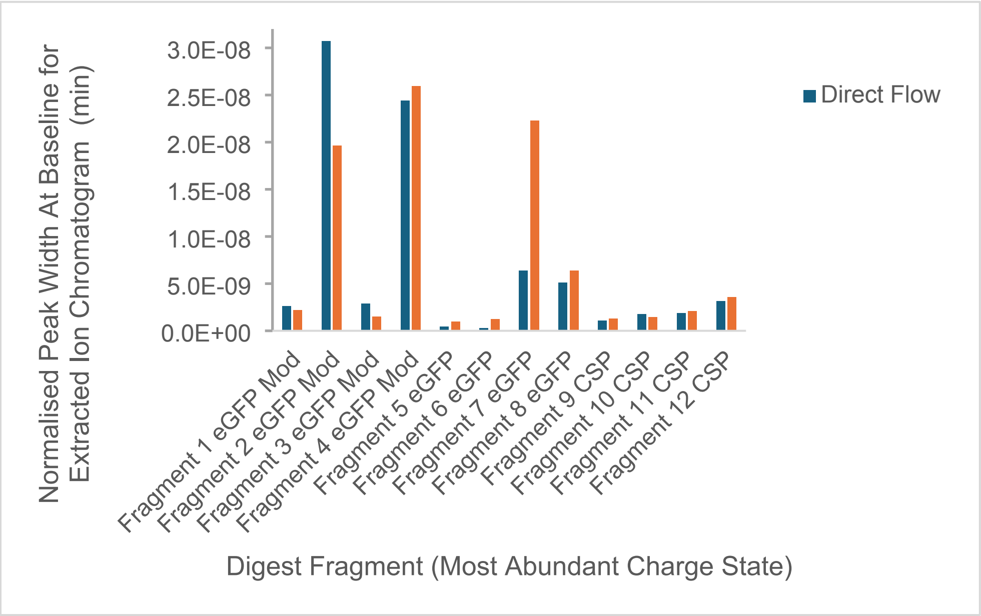

Supporting Figure S2. Comparative analysis of oligoribonucleotide fragments generated using online and offline partial RNase T1 digests. A) MS intensity of most abundant charge state for a range of eGFP and CSP mRNA RNase fragments. B) Peak width at baseline for each fragment divided by the MS peak area for a range of eGFP and CSP mRNA RNase fragments .

Supporting Table S2. Comparison of mRNA % sequence coverage for eGFP mRNA analysed using online partial RNase T1 digestion across varying RNase T1 column temperatures. Flow rate was fixed at 50 µL/min. Sequence coverages obtained from searching the LC MS/MS data against a randomised RNA sequence of the same size and GC content is highlighted. All sequence coverage values are based on unique oligoribonucleotide identifications.

Digestion Column Temperature (°C)	mRNA sequence coverage (%)	Randomized RNA sequence coverage (%)
5	95.7	2.7
15	99.1	2.7
25	94.2	9.3
35	80.5	4.2
45	49.9	0.0

Supporting Table S3. mRNA % sequence coverage for CSP mRNA analysed using online partial RNase T1 digestion across varying temperatures of the RNase T1 column. Flow rate was fixed at 20 µL/min. Sequence coverages obtained from searching the LC MS/MS data against a randomised RNA sequence of the same size and GC content are highlighted. All sequence coverage values are based on unique oligoribonucleotide identifications.

Digestion Column Temperature (°C)	mRNA sequence coverage (%)	Randomised RNA sequence coverage (%)
5	56.6	5.2
15	66.1	5.6
25	61.6	6
35	25.2	1
45	7.6	0.5

Supporting Table S4. mRNA % sequence coverage for eGFP mRNA analysed using online partial RNase T1 digestion using varying flow rates across the RNase T1 column. Temperature was fixed at 25 °C. Sequence coverages obtained from searching the LC MS/MS data against a randomised RNA sequence of the same size and GC content are highlighted. All sequence coverages are based on unique oligoribonucleotide identifications.

Flow Rate (µL/min)	mRNA sequence coverage (%)	Randomised RNA sequence coverage (%)
10	69.5	0.0
30	76.0	0.0
40	91.3	5.9
50	92.6	8.8
60	94.9	7.6
70	96.1	9.1
30	76.0	0.0

Supporting Table S5. mRNA % sequence coverage for CSP mRNA analysed using online partial RNase T1 digestion using a range of flow rates across the RNase T1 column. Temperature was fixed at 20 °C. Sequence coverages obtained from searching the LC MS/MS data against a randomised RNA sequence of the same size and GC content are highlighted. All sequence coverage values are based on unique oligoribonucleotide identifications.

Flow Rate (µL/min)	mRNA sequence coverage (%)	Randomised RNA sequence coverage (%)
10	45.0	10.3
20	60.4	10.2
30	64.5	9.5
40	64.9	9.1
50	81.8	10.2

Supporting Table S6. Comparison of mRNA % sequence coverage for eGFP and CSP mRNA analysed using online partial RNase T1 digest in Loop Capture and Direct Flow 2D LC. Sequence coverages obtained from searching the LC MS/MS data against a randomised RNA sequence of the same size and GC content are highlighted. All sequence coverage values are based on unique oligoribonucleotide identifications.

**mRNA**	**Loading (µg)**	**Sequence Coverage (%)** ***Loop Capture***	**Sequence coverage randomized RNA (%)** ***Loop Capture***	**Sequence Coverage (%)** ***Direct Flow***	**Sequence coverage randomized RNA (%)** ***Direct Flow***
eGFP	10	93.2	2.3	94.3	0.0
eGFP m1Ψ	11	90.7	0.0	78.3	0.0
CSP	18	72.6	8.5	69.5	3.8
CSP m1Ψ	19	60.0	4.1	N/A	N/A

Supporting Table S7. % abundance of oligoribonucleotide fragments identified with 2’,3’ cyclic phosphate and 3’ phosphate termini for eGFP and CSP mRNA analysed using offline and online RNase T1 digests. Online digests for eGFP mRNA were performed at 25 °C, 50 µL/min and CSP mRNA performed at 20 °C, 20 µL/min.

	2',3' cyclic phosphate (%)	3' phosphate (%)
Offline eGFP mRNA	100.0	0.0
Online eGFP mRNA	84.3	15.7
Offline CSP mRNA	99.0	1.0
Online CSP mRNA	86.5	13.5

Supporting Table S8. Comparison of average MS peak area and mRNA % sequence coverage. eGFP mRNA was analysed using online and offline partial RNase T1 digests. Online digests for eGFP mRNA were performed at 25 °C, 50 µL/min. All sequence coverage values are based on unique oligoribonucleotide identifications.

RNase T1 Digestion Method	eGFP mRNA (µg)	Average MS Area	mRNA Sequence Coverage (%)
Offline	10.0	3.26E+06	91.3
Online	10.0	5.84E+06	94.8
Offline	1.0	6.02E+05	83.3
Online	1.0	7.97E+05	72.9
